# Obstructive Sleep Apnea Activates HIF-1 in a Hypoxia Dose-Dependent Manner in HCT116 Colorectal Carcinoma Cells

**DOI:** 10.3390/ijms20020445

**Published:** 2019-01-21

**Authors:** Chloe-Anne Martinez, Bernadette Kerr, Charley Jin, Peter A. Cistulli, Kristina M. Cook

**Affiliations:** 1 Charles Perkins Centre, Faculty of Medicine and Health, Northern Clinical School, The University of Sydney, Sydney NSW 2006, Australia; cmar9054@uni.sydney.edu.au (C.-A.M.); bernadette.kerr@sydney.edu.au (B.K.); cjin9006@uni.sydney.edu.au (C.J.); peter.cistulli@sydney.edu.au (P.A.C.); 2 Department of Respiratory and Sleep Medicine, Royal North Shore Hospital, Sydney 2065, Australia

**Keywords:** intermittent hypoxia, obstructive sleep apnea, HIF-1, cancer, hypoxia

## Abstract

Obstructive sleep apnea (OSA) affects a significant proportion of the population and is linked to increased rates of cancer development and a worse cancer outcome. OSA is characterized by nocturnal intermittent hypoxia and animal models of OSA-like intermittent hypoxia show increased tumor growth and metastasis. Advanced tumors typically have regions of chronic hypoxia, activating the transcription factor, HIF-1, which controls the expression of genes involved in cancer progression. Rapid intermittent hypoxia from OSA has been proposed to increase HIF-1 activity and this may occur in tumors. The effect of exposing a developing tumor to OSA-like intermittent hypoxia is largely unknown. We have built a cell-based model of physiological OSA tissue oxygenation in order to study the effects of intermittent hypoxia in HCT116 colorectal cancer cells. We found that HIF-1α increases following intermittent hypoxia and that the expression of HIF-target genes increases, including those involved in glycolysis, the hypoxic pathway and extracellular matrix remodeling. Expression of these genes acts as a ‘hypoxic’ signature which is associated with a worse prognosis. The total dose of hypoxia determined the magnitude of change in the hypoxic signature rather than the frequency or duration of hypoxia-reoxygenation cycles per se. Finally, transcription of *HIF1A* mRNA differs in response to chronic and intermittent hypoxia suggesting that HIF-1α may be regulated at the transcriptional level in intermittent hypoxia and not just by the post-translational oxygen-dependent degradation pathway seen in chronic hypoxia.

## 1. Introduction

Obstructive sleep apnea (OSA) is increasingly prevalent, affecting 13% of men and 6% of women [1], and has recently been linked to an increased risk of cancer and cancer mortality [2,3]. A prominent characteristic of OSA is the cyclical systemic intermittent hypoxia generated by upper airway obstruction during sleep. Animals exposed to OSA-like intermittent hypoxia have increased tumor growth, invasion and metastasis [4,5,6,7,8], implying that intermittent hypoxia is driving many of the pathological effects of OSA. 

A separate phenomenon, known as chronic tumor hypoxia, is linked to poor cancer outcomes [3]. Chronic tumor hypoxia activates the transcription factor hypoxia inducible factor-1 (HIF-1), which controls the expression of many cancer-related genes, including those involved in glycolysis (e.g., *LDHA,* lactate dehydrogenase A; *HK2,* hexokinase 2), metastasis (e.g., *P4HA1*, prolyl 4-Hydroxylase Subunit Alpha 1; *P4HA1*, prolyl 4-Hydroxylase Subunit Alpha 1) and angiogenesis (e.g., *ADM*, adrenomedullin; *ANGPTL4,* angiopoietin-like 4) [9]. HIF-1 plays an important role in cancer progression and patients with increased HIF-1 generally have a worse cancer prognosis [3,9]. 

**Figure 1:**
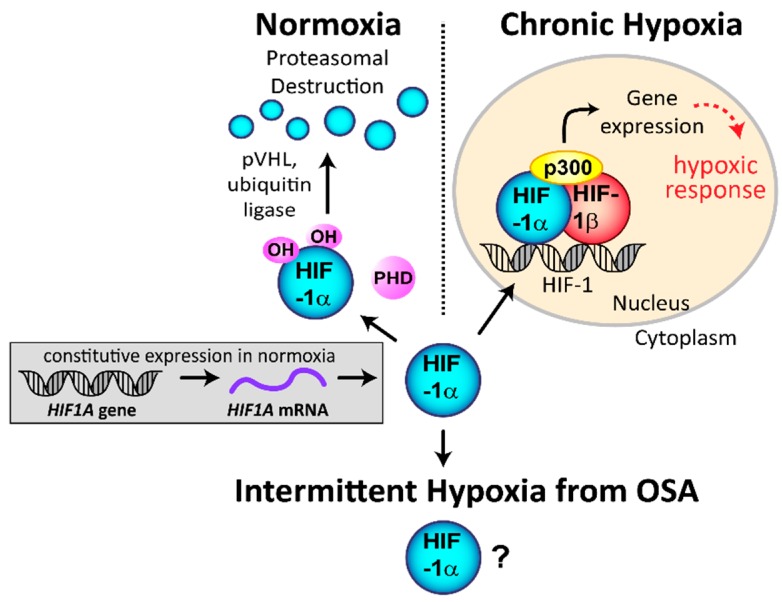
Regulation of hypoxia inducible factor-1 α (HIF-1α) stability and HIF-1 transactivation under normoxia and hypoxia. Under normal oxygen conditions, the *HIF1A* gene is constitutively transcribed to *HIF1A* mRNA, which is then translated to HIF-1α protein. In normoxia and the presence of oxygen, the HIF-1α protein is rapidly hydroxylated by the prolyl hydroxylases (PHDs) enzymes, which enables binding of von Hippel–Lindau tumor suppressor protein (pVHL) and the ubiquitin ligase, leading to proteasomal degradation. Under chronic hypoxia, hydroxylation no longer occurs, enabling HIF-1α to move into the nucleus and form the active HIF transcription complex. The regulation and activation of HIF-1α under intermittent hypoxia from obstructive sleep apnea (OSA) is not entirely clear. Modified from Hunyor et al. [3].

HIF-1 is composed of HIF-1α and HIF-1β subunits and the transcriptional coactivator p300/CBP (Figure 1). Activity of the entire complex is regulated through the HIF-1α subunit in a post-translational, oxygen-dependent process. *HIF1A* is constitutively transcribed and translated into HIF-1α protein during normoxia. The HIF-1α protein is then continuously degraded through an oxygen-dependent degradation process via the prolyl hydroxylases (PHDs). The PHDs hydroxylate prolines within HIF-1α using 2-oxoglutarate and oxygen as substrates, and iron and ascorbate as cofactors [10]. HIF-1α proline hydroxylation enables the von Hippel–Lindau tumor suppressor protein (pVHL) to bind to HIF-1α and link to the ubiquitin ligase complex, targeting it for degradation by the proteasome. Under hypoxia, degradation is impaired, allowing the constitutively made HIF-1α protein to translocate into the nucleus and form the active HIF complex (Figure 1) [3]. Under sustained hypoxia, transcription of *HIF1A* decreases, leading to transient stabilization of HIF-1α protein, while its mRNA is suppressed [11]. This mechanism acts as negative feedback to prevent over-activation of HIF-1.

While longer periods of intermittent hypoxia (hours and days) have been shown to increase HIF-1 activity [12,13], it is not clear if rapid OSA-like intermittent hypoxia (minutes) is capable of inducing HIF-1 activity and which tissues are affected. Few studies have examined whether longer periods of intermittent hypoxia are biologically equivalent to rapid cycles of intermittent hypoxia. There is a paucity of data to show if HIF-1α is stabilized by OSA-like intermittent hypoxia in tumors, mainly because rapidly changing dissolved oxygen in cell culture is difficult. The few that do exist demonstrate conflicting results [14,15]. This is likely due to the different cell types and intermittent hypoxia protocols employed. Similarly, it is unclear if HIF-1α stabilization leads to HIF-1 activity and which genes are expressed [15]. HIF-1α is increased by rapid intermittent hypoxia in non-cancerous cell lines, including cells of the carotid body [16], and only in one of two studies using endothelial cells [15,17]. HIF-1 was not activated by intermittent hypoxia using HeLa cervical cancer cells, though shorter total exposures to intermittent hypoxia (30 min–4 h) were used as compared to usual sleep durations [14]. The relationship between increased levels of HIF-1α, transactivation and gene expression is poorly described in tumor cells exposed to OSA. 

**Figure 2:**
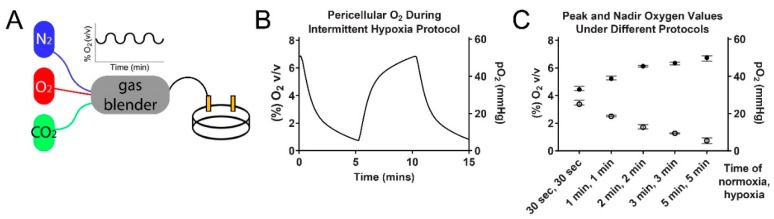
Building a model of OSA-like intermittent hypoxia. (**A**) The model for exposing cells to intermittent hypoxia. A programmable gas blender controls oxygen delivery to a small chamber containing cells grown on oxygen permeable dishes. (**B**) Pericellular oxygen levels during the administered intermittent hypoxia protocol. (**C**) The peak and nadir pericellular oxygen measurements using different lengths of normoxia and hypoxia in the gas phase (normoxia set to 12% oxygen and hypoxia set to 0% oxygen). Average values ± range.

Our first aim was to develop a cellular model of intermittent hypoxia that closely replicates the in vivo oxygen changes in a tumor exposed to OSA (Figure 2). We used this model to study the effects of intermittent hypoxia in tumor cells and determine if the HIF-1 pathway is affected. We found that HIF-1α protein does increase in HCT116 colorectal cancer cells exposed to OSA-like oxygen changes, and it alters gene and protein expression in pathways regulating glycolysis, hypoxia and extracellular matrix remodeling. We also identified that there are different HIF regulatory pathways at play when studying the separate effects of chronic and intermittent hypoxia.

## 2. Results

### 2.1. HIF-1α Protein Increases in Rapid Intermittent Hypoxia

**Figure 3:**
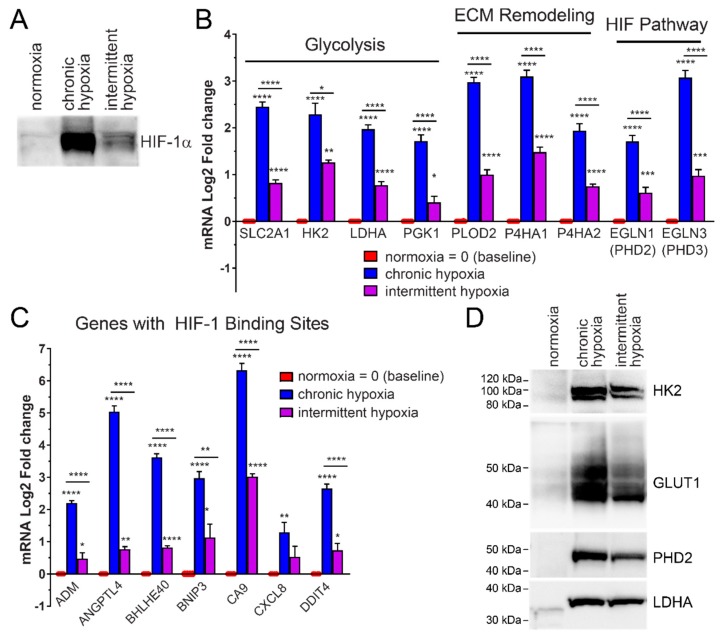
HIF-1α protein increases in HCT116 cells exposed to rapid intermittent hypoxia. Increased HIF-1α increases HIF-1 transactivation, leading to increased expression of HIF-1 target genes at both the mRNA and protein level. (**A**) Rapid intermittent hypoxia increases HIF-1α protein after 6 h of exposure. (**B**) Rapid intermittent hypoxia increases the mRNA expression of genes involved in glycolysis, extracellular matrix remodeling and the HIF pathway after 18 h of exposure. (**C**) Additional genes with known hypoxia response elements (HRE) binding sites for HIF-1 were measured and all tested genes showed an increase in both chronic hypoxia and intermittent hypoxia after 18 h. All values were normalized to normoxic expression levels, which is equivalent to 0 on the Log2 scale. Results are the mean ± SEM of independent experiments run in duplicate (*n* ≥ 3). (**D**) The increase in mRNAs is also reflected in an increase in the protein levels of Glut1, HK2, LDHA and PHD2. * *p* < 0.05, ** *p* < 0.01, *** *p* < 0.001 and **** *p <* 0.0001.

To determine if HIF-1α protein is stabilized in tumor cells by OSA-like cycles of intermittent hypoxia, HCT116 colorectal cancer cells were exposed to intermittent hypoxia for 6 h similar to a typical night of sleep. Intermittent hypoxia led to HIF-1α stabilization as compared to normoxia (Figure 3A). HIF-1α protein levels in intermittent hypoxia were also compared to the same time period of chronic hypoxia. Following 6 h of chronic hypoxia, there was a significant increase in the amount of HIF-1α nuclear protein present as compared to normoxia. Chronic hypoxia led to the greatest increase in HIF-1α protein (Figure 3A).

### 2.2. Rapid Intermittent Hypoxia Increases the Expression of Genes Associated with a Poor Cancer Prognosis Including Those Involved in Glycolysis, ECM Remodeling and the HIF Pathway

Expression of glycolytic enzymes, including hexokinase 2 (*HK2*), lactate dehydrogenase A (*LDHA*) and phosphoglycerate kinase 1 (*PGK1*) and the glucose transporter, Glut1, are induced by chronic hypoxia (Figure 3B) and this is mediated by HIF-1 [18]. To test if the HIF-1α increase from intermittent hypoxia led to expression of glycolysis genes, HCT116 cells were exposed to 18 h of different oxygen conditions. We found a significant increase in the mRNA expression of HIF-1-target genes *HK2*, *LDHA*, *PGK1*, and Glut1 (*SLC2A1*), in cells exposed to intermittent hypoxia. These mRNA increases were significantly more than in normoxia but less than in chronic hypoxia (0.5%) (Figure 3B).

To determine if HIF transactivation was occurring in pathways other than glycolysis, we measured expression of genes with known hypoxia response elements (HRE) binding sites involved in extracellular matrix remodeling (ECM) and metastasis [19], as well as the HIF pathway itself. mRNA expression of the ECM-related genes, lysyl hydroxylase *PLOD2*, and prolyl hydroxylases *P4HA1* and *P4HA2*, increased in intermittent and chronic hypoxia (Figure 3B). mRNA expression of the HIF-regulatory prolyl hydroxylases, PHD2 (*EGLN1*) and PHD3 (*EGLN3*) [20,21,22] also significantly increased following exposure to intermittent and chronic hypoxia (Figure 3B).

We saw increased mRNA expression of *ADM* (adrenomedullin), *ANGPTL4* (angiopoietin-like 4), *BHLHE40* (Class E basic helix-loop-helix protein 40, also known as Dec1), *BNIP3* (BCL2/adenovirus E1B 19 kDa protein-interacting protein 3), *CA9* (carbonic anhydrase IX), *CXCL8* (interleukin 8) and *DDIT4* (DNA-damage-inducible transcript 4), all genes with confirmed HRE binding sites in their promoters [23] and roles in cancer progression (Figure 3C).

We also measured how the increased mRNA expression affected the protein levels for specific genes. Western blotting was used to examine protein levels of HK2, Glut1, LDHA and PHD2. Rapid intermittent hypoxia significantly increased all of the HIF-target proteins examined (Figure 3D).

Importantly, increased expression of many of these genes, including *SLC2A1*, *HK2*, *CA9*, *LDHA*, *BNIP3*, *PGK1*, *P4HA1* and *ADM* have been proposed as a hypoxic signature or ‘metagene’ which has negatively prognostic value for a wide range of cancers [24]. Intermittent hypoxia mimicking OSA was sufficient to induce the expression of multiple prognostic genes.

### 2.3. siRNA-Mediated Knockdown of HIF-1α Decreases Expression of Glycolysis, ECM Remodeling and HIF Pathway Genes in Intermittent Hypoxia

**Figure 4:**
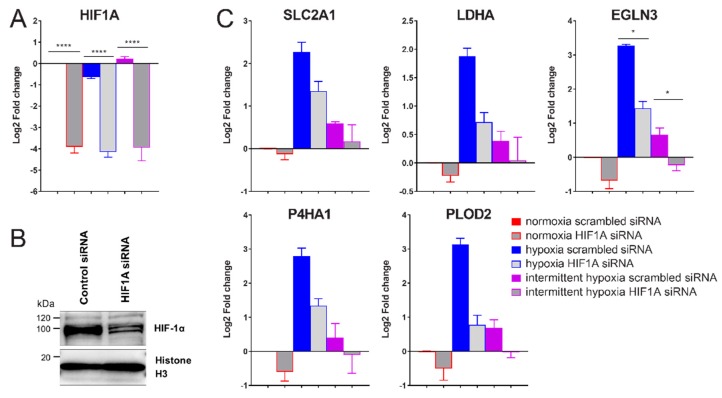
*HIF1A* siRNA mediated knockdown reduces intermittent hypoxia-driven increases in gene expression. (**A**) qPCR data showing the effect of *HIF1A* siRNA on *HIF1A* mRNA expression. All values were normalized to normoxia expression levels with scrambled (negative) control siRNA, which is set to 0 on the Log2 scale. (**B**) HIF-1α western blot of HCT116 cells in hypoxia treated with either scrambled siRNA or *HIF1A* siRNA. (**C**) HIF-1α target gene expression following *HIF1A* siRNA transfection under normoxic, chronic hypoxic or intermittent hypoxic conditions. Results are the mean ± SEM of independent experiments run in duplicate (*n* ≥ 3). * *p* < 0.05 and **** *p* < 0.0001.

To verify if the increase in HIF-1α during intermittent hypoxia was directly increasing target gene expression through increased HIF-1 transactivation, HCT116 cells were transfected with *HIF1A* siRNA or scrambled control siRNA and exposed to normoxia, intermittent hypoxia and chronic hypoxia as before. The *HIF1A* siRNA led to a 95%, 92% and 95% decrease in *HIF1A* mRNA in normoxia, chronic hypoxia and intermittent hypoxia respectively, 48 h after transfection (Figure 4A). HIF-1α protein decreases were verified using western blotting following siRNA transfection (Figure 4B).

Knockdown of *HIF1A* expression led to a significant decrease in all measured hypoxia-induced genes (Figure 4C) in both intermittent and chronic hypoxia. This means HIF-1α is responsible for the increased HIF-1 activity and target gene expression seen in Figure 3B.

### 2.4. Intermittent Hypoxia Has a Hypoxia Dose-Dependent Effect for Expression of the “Hypoxic Signature”

**Figure 5:**
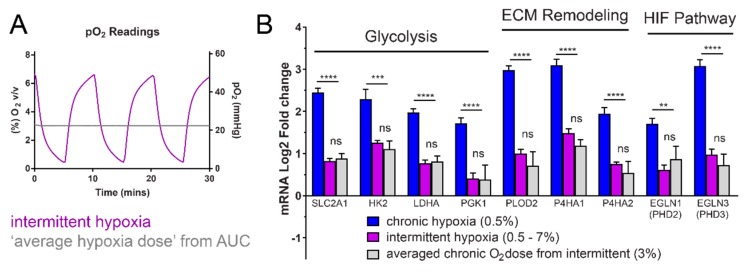
Hypoxia dose-dependent effect of intermittent hypoxia. (**A**) The area under the intermittent hypoxia curve (purple) was calculated using the measured pericellular oxygen values, to determine the ‘average’ dose of hypoxia (grey). (**B**) The equivalent chronic hypoxia dose at 3% leads to the same increase in HIF-target gene expression. The expression data from Figure 2B was compared to expression under 3% hypoxia conditions. 3% oxygen is the ‘averaged hypoxic dose’ equivalent to the same amount of oxygen given in one full cycle of intermittent hypoxia, but administered as a constant dose. The average hypoxia dose was calculated using the area under the curve from oxygen measurements in (**A**). Horizontal bars indicate comparison between chronic hypoxia (0.5%) and averaged intermittent hypoxia dose (3%). Results are the mean ± SEM of independent experiments run in duplicate (*n* ≥ 3). ** *p* < 0.01, ns = not significant, *** *p* < 0.001 and **** *p* < 0.0001.

It is unclear if the effects of intermittent hypoxia result from a dose-dependent-hypoxia effect or from repetitive hypoxia-reoxygenation periods. To test whether the reoxygenation cycles were important for this expression, we calculated the average dose of hypoxia based on the area under the curve from our pericellular oxygen readings (Figure 5A). We found that expression of the measured HIF-1 target genes was the same between the averaged, chronic 3% oxygen (22 mmHg) dose of hypoxia and the intermittent hypoxia fluctuating between 0.5–7% (~4–50 mmHg) oxygen (Figure 5B). These results indicate that for expression of *SLC2A1*, *HK2*, *LDHA*, *PGK1*, *PLOD2*, *P4HA1*, *P4HA2*, *EGLN1* and *EGLN3*, cycles of reoxygenation are less important than the total dose of hypoxia which determines the magnitude of expression for the measured HIF-1 target genes.

### 2.5. Chronic Hypoxia Decreases HIF1A mRNA, While Intermittent Hypoxia Increases HIF1A mRNA Synthesis

Under prolonged hypoxia, increased levels of HIF-1α protein upregulate negative feedback systems that prevent excessive HIF-1 activity [25]. The resulting effect is suppression of HIF1A gene transcription, therefore decreasing HIF-1α mRNA and protein over time.

**Figure 6:**
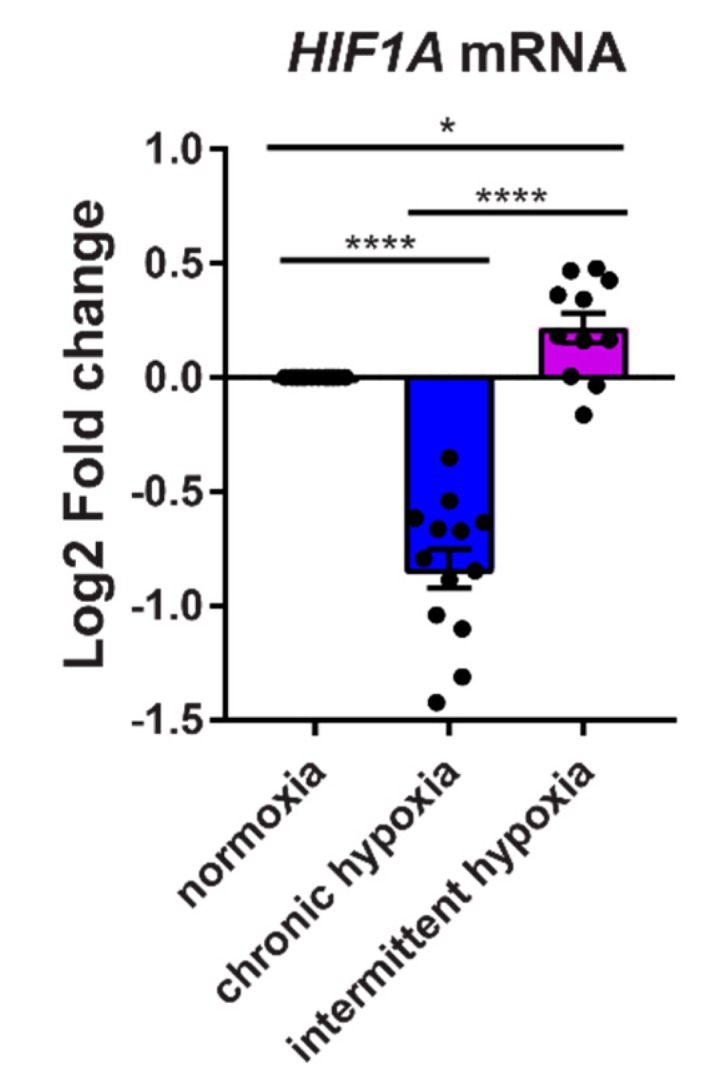
Expression of *HIF1A* transcription differs between chronic and intermittent hypoxia. HCT116 cells were exposed to normoxia, chronic hypoxia and intermittent hypoxia for 18 h before measuring *HIF1A* mRNA expression. Results are the mean ± SEM of independent experiments run in duplicate (*n* ≥ 11). * *p* < 0.05 and **** *p* < 0.0001.

To determine if HIF1A transcription is suppressed following intermittent hypoxia exposure, we exposed HCT116 cells to 18 h of normoxia, chronic hypoxia and intermittent hypoxia. In agreement with previous studies [11], we found that after 18 h of chronic hypoxia, HIF1A mRNA significantly decreases below normoxic levels (Figure 6). In intermittent hypoxia, HIF1A transcription significantly increases above normoxia and chronic hypoxia. This implies that regulation of HIF-1α protein levels in intermittent hypoxia may not be regulated solely at the post-translational level but also at the transcriptional level.

## 3. Discussion

Obstructive sleep apnea is typically associated with cardiovascular disease and stroke but has recently been linked to a higher rate of cancer incidence and worse cancer outcome [3]. Much of the research into molecular drivers of this disease has focused on the inflammatory effects from intermittent hypoxia. However, given the link between chronic hypoxia in tumors and worse cancer outcomes, there is a recent interest in the effects of hypoxia from OSA.

To our knowledge, this is the first study of OSA that uses physiologically relevant oxygen fluctuations to examine whether HIF is activated in tumor cells. Currently, there are no standardized protocols defining a pattern of intermittent hypoxia that equates to OSA. The duration, severity, and frequency of intermittent hypoxia varies widely, contributing to heterogeneity of pre-existing results. ‘Normoxia’ and ‘hypoxia’ are relative values and physiologically relevant values are tissue-specific. In our model, we have tried to use oxygen levels that correspond to what a small, developing tumor would experience in vivo, based on measured pO_2_ values from a study conducted in mice [6], as well as pO_2_ measurements in healthy tissues [3].

We found that intermittent hypoxia mimicking OSA increases HIF-1α and expression of HIF-1 target genes, resulting in a ‘hypoxic signature’ that is associated with a worse cancer prognosis [24]. Intermittent hypoxia mimicking OSA was enough of a hypoxic stimulus to induce expression of the ‘hypoxic signature’ when compared to physiological normoxia mimicking the microenvironment of an early stage tumor. This implies that OSA may be sufficient to induce HIF-1 activation in an early stage tumor that has not yet developed regions of chronic hypoxia. Given the strong correlation between the presence of HIF-1 and a poor cancer outcome, early activation of HIF-1 during cancer development may have significant adverse sequelae.

While the negative effects of chronic tumor hypoxia are well known, it is a phenomenon that occurs after the initial tumor develops past a certain size. In contrast, OSA patients are chronically exposed to intermittent hypoxia both before and after tumorigenesis. A single cell with tumorigenic potential is exposed to OSA night after night, prior to development of chronic tumor hypoxia. Exposure to intermittent hypoxia and activation of HIF-1 early in tumorigenesis may be driving the tumor growth and aggressiveness seen in melanoma patients with OSA and in animal models of OSA [4,5,6,26].

Interestingly, our results showed that for the hypoxic signature, expression levels depended on a dose-dependent effect of hypoxia. Reoxygenation-hypoxia cycles were not necessary to induce gene expression, as an averaged oxygen dose based on the area under the curve from our intermittent hypoxia cycles resulted in similar gene expression patterns. These results are clinically relevant, with a previous epidemiological study showing that there was a dose-dependent effect of nocturnal hypoxemia on cancer mortality [27]. As hypoxemia increased, the chances of dying from cancer increased and our results suggest this may be due to a HIF dose-dependent effect.

siRNA mediated knockdown of HIF-1α reduced expression of the hypoxic gene expression signature under intermittent hypoxia, confirming HIF-1 is responsible for the observed increases. This suggests that OSA-mediated increases in HIF activity may be amenable to pharmacological inhibition and there are many HIF inhibitors being developed by ourselves and others [18,28,29,30,31].

**Figure 7:**
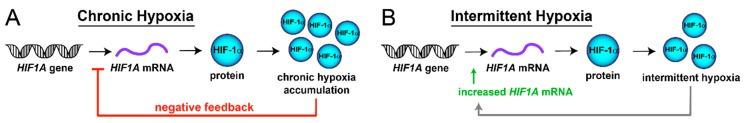
Regulation of *HIF1A* transcription differs between chronic and intermittent hypoxia. The *HIF1A* gene is expressed into *HIF1A* mRNA, which is translated into HIF-1α protein. The stability of HIF-1α protein is regulated in an oxygen-dependent post-translational manner. (**A**) Increased HIF-1α protein stabilization in chronic hypoxia leads to the activation of negative feedback systems which suppress *HIF1A* gene expression. (**B**) HIF-1α protein is also stabilized in intermittent hypoxia, though to a lower level as compared to chronic hypoxia, and this does not activate negative feedback loops that suppress transcription of *HIF1A*.

HIF activity is typically regulated by the oxygen-dependent degradation of HIF-1α in normoxia. In the absence of oxygen, HIF-1α is stabilized and can move into the nucleus to form the active transcription factor. However, there are other mechanisms that can regulate HIF activity, including changes in the transcription of the *HIF1A* gene, which can affect the amount of HIF-1α protein in the cell. In chronic hypoxia, transcription of *HIF1A* mRNA is suppressed through a negative feedback system which acts to prevent overaction of HIF-1 [25]. In contrast to this, we found that in intermittent hypoxia, *HIF1A* transcription significantly increases above that seen in normoxia and chronic hypoxia (Figure 6). This indicates that HIF-1α protein levels in intermittent hypoxia may be regulated partially at the transcriptional level and not just by the canonical, post-translational oxygen-dependent degradation pathway. The increased HIF-1α protein from intermittent hypoxia does not appear to activate the negative feedback systems which lead to suppressed *HIF1A* gene transcription in chronic hypoxia [25] (Figure 7A,B). It is possible that a ‘critical mass’ of HIF-1α protein is required to activate the negative feedback mechanisms, which is not reached in intermittent hypoxia, yet is still sufficient to induce expression of the ‘hypoxic signature’.

We have developed a model for studying the effects of intermittent hypoxia from OSA that overcomes several limitations from previous studies. Our model uses shorter periods of hypoxia, which more closely represent the rapid periods of hypoxia seen in OSA patients. Our model does not cause shear stress from changing cell culture media to rapidly alter dissolved pO_2_. We are also the first to use dissolved pO_2_ values that mimic actual tissue oxygenation based on mouse models.

While the cells in our model are cultured on membranes that are highly permeable to oxygen, equilibrium with the gas phase is not instantaneous. This meant that our study has the limitation of using 5 min periods of hypoxia, which were required to reach the peak and nadir oxygen values seen in vivo. As improvements are made in oxygen-permeable materials, we expect that the cellular models of rapid intermittent hypoxia will be capable of even shorter periods of hypoxia.

In summary, we have found that HIF-1α activity increases in HCT116 colorectal tumor cells following exposure to intermittent hypoxia mimicking OSA. This increases the expression of HIF-target gene and protein expression in pathways associated with cancer progression.

## 4. Materials and Methods

### 4.1. Developing a Cellular Model of Intermittent Hypoxia

To overcome the difficulties in generating rapidly changing oxygen levels, we developed a model similar to Minoves et al. [15] and adapted it to more closely mimic tumor oxygenation in vivo. Cells are grown on oxygen permeable membranes enabling oxygen to be sourced directly through the membrane from the gas phase.

#### 4.1.1. Defining Normoxia

Many studies of physiological “intermittent hypoxia” in OSA use room air for peak oxygenation (21% *v*/*v*, 155 mmHg). Blood/tissue oxygenation has been measured under normal ventilatory conditions and values are much lower, typically between 30–100 mmHg [3]. We selected physiological “normoxia” to reflect tissue oxygenation near major blood vessels (89 mmHg, 12% *v*/*v*).

Defining chronic hypoxia. Most large tumors have “hypoxic” regions with much lower oxygen values than their corresponding healthy tissue. Based on previous data, we selected 4 mmHg (0.5% *v*/*v*) oxygen to mimic chronic tumor hypoxia [32,33].

#### 4.1.2. Defining Intermittent Hypoxia

Data is lacking to accurately define the partial pressure of oxygen (pO_2_) in human tissues during OSA events. However, several studies have directly measured pO_2_ in select tissues using an animal model of OSA [6,34,35,36,37], including one type of tumor (subcutaneous melanoma) [6]. The pO_2_ within the tumor during “apneas” ranged from ~5 mmHg to ~50–55 mmHg, and the nadir and peak values we selected for our model (~5–50 mmHg) closely match those from the study [6].

#### 4.1.3. Protocol

HCT116 colorectal carcinoma cells were maintained as before [38] and grown in 2-well oxygen-permeable dishes (1.5 × 10^5^ cells/cm^2^ (Coy Laboratory, Grass Lake, MI, USA)) prior to experiments. Before normoxia/hypoxia exposure, cells were switched to media with 0.5% FBS. Total gas flow from mixer was set to 500 mL/min. For the normoxic and hypoxic controls, two pre-mixed gas canisters (89 mmHg oxygen for normoxia and 4 mmHg oxygen for chronic hypoxia) were used. 5% CO_2_ (38 mmHg) was included in all gas mixes. Dishes were placed in custom-made acrylic chambers and connected to a programmable gas mixer attached to nitrogen, oxygen and carbon dioxide gas sources (Gas Blender 100, MCQ Instruments, Rome, Italy) (Figure 2A). We found that by using 5 min periods of 59 mmHg oxygen (gas phase), followed by 5 min of 0 mmHg oxygen (gas phase), we could generate pO_2_ curves consistently fluctuating between ~5–50 mmHg oxygen in the pericellular media (see Figure 2B). Altering the lengths of “normoxia” and “hypoxia” gave different peak and nadir oxygen values (Figure 2C). Exposures to hypoxic conditions were for either 6 h (HIF-1α protein assessment) or 18 h (HIF-target gene expression assessment) based on a study indicating different peaks for HIF-1α protein and HIF-target expression [39].

### 4.2. Western Blotting

To measure HIF-1α, cells were exposed to oxygen conditions for 6 h, as HIF-1α protein levels peak after approximately 6 h of hypoxia exposure and then gradually decline [39,40]. For downstream HIF-1 target genes, cell lysates were generated following 18 h of normoxia, chronic hypoxia and intermittent hypoxia (near the peak of HIF-1 target gene expression [39]). Lysates were prepared using RIPA buffer containing protease inhibitors (Roche complete, Sigma-Aldrich, Castle Hill, NSW Australia) and resolved using SDS-PAGE and western blotting as before [41]. Antibodies were as follows: HIF-1α (NB100-479, Novus), Glut1 (ab115730, Abcam, Cambridge, MA, USA), hexokinase 2 (TA325030, Origene), Histone H3 (ab1791, Abcam), PHD2 (4835, Cell Signaling Technologies, Danvers, MA, USA) and LDHA (3582T, Cell Signaling Technologies).

### 4.3. Quantitative Real-Time PCR

Following 18 h of normoxia, chronic hypoxia and intermittent hypoxia (near the peak of HIF-1 target gene expression [41]), total RNA was extracted using the RNeasy mini kit (Qiagen, Hilden, Germany). RNA concentration was determined using a NanoDrop^®^ (Molecular Devices, San Jose, CA, USA). RNA integrity was verified using an RNA bleach gel [42]. Purified RNA (2 μg) was reverse transcribed using the High Capacity cDNA Reverse Transcription Kit (ThermoFisher, North Ryde, NSW, Australia).

For qPCR, TaqMan Gene Expression Assays and TaqMan Gene Expression Master mix were purchased from ThermoFisher Scientific. qPCR was performed using a QuantStudio 7 Flex Real-Time PCR System. Relative gene expression was normalized to eukaryotic 18S rRNA and fold-change was calculated using the ΔΔ*C*_t_ method.

TaqMan Primers (Assay ID) used for qPCR (ThermoFisher Scientific) with gene name followed by protein name in parentheses. SLC2A1 (Glut1) (Hs00892681_m1), HK2 (hexokinase 2) (Hs00606086_m1), LDHA (lactate dehydrogenase A) (Hs01378790_g1), PGK1 (phosphoglycerate kinase 1) (Hs00943178_g1), PLOD2 (Procollagen-Lysine,2-Oxoglutarate 5-Dioxygenase 2) (Hs01118190_m1), P4HA1 (Prolyl 4-Hydroxylase Subunit Alpha 1) (Hs00914594_m1), P4HA2 (Prolyl 4-hydroxylase subunit alpha-2) (Hs00990001_m1), HIF1A (HIF-1α) (Hs00153153_m1), EGLN1 (prolyl hydroxylase domain 2) (Hs00254392_m1), EGLN3 (prolyl hydroxylase domain 3) (Hs00222966_m1), ADM (Adrenomedullin) (Hs00181605_m1), ANGPTL4 (Angiopoietin-like 4) (Hs00211522_m1), BHLHE40 (Basic Helix-Loop-Helix Family Member E40) (Hs00186419_m1), CXCL8 (interleukin-8) (Hs00174103_m1), CA9 (carbonic anhydrase IX) (Hs00154208_m1), DDIT4 (DNA Damage Inducible Transcript 4) (Hs00430304_g1), and 18S (Hs03003631_g1).

### 4.4. Pericellular pO_2_ and Calculating the Area under the Curve

The pericellular pO_2_ (dissolved O_2_) was measured during intermittent hypoxia using an oxygen PSt7 sensor with temperature compensation connected to an OXY1 single channel fiber oxygen transmitter (PreSens, Regensburg, Germany). Pericellular oxygen levels in media were measured by placing electrode directly on the gas permeable membrane in culture media where cells were growing. Readings were recorded using PreSens measurement Studio 2.

The average dose of hypoxia from intermittent hypoxia was calculated from the area under the curve from our pericellular oxygen readings. The average equivalent dose of hypoxia from intermittent hypoxia was found to be 22 mmHg and cells were exposed to this as a chronic dose for similar times as normoxia, chronic and intermittent hypoxia.

### 4.5. siRNA and HIF-1α Knockdown

HIF-1α Flexitube GeneSolution siRNA (GS3091) and AllStars Negative control siRNA (SI03650318) were used (Qiagen). HCT116 cells were transfected with siRNA using a reverse transfection method with HiPerfect (Qiagen). 30 h after transfection, media was replaced with McCoy’s 5A (modified) media containing 0.5% FBS, penicillin/streptomycin and glutamax and exposed to normoxia, chronic and intermittent hypoxia for 18 h as described. Cells were harvested for qRT-PCR or western blotting.

### 4.6. Statistical Analysis

Data are expressed as mean ± SEM from independent experiments. Statistical analysis was performed using a One-Way ANOVA function with multiple comparisons follow-up test (comparing the mean of each column with the mean of every other column). * *p* < 0.05, ** *p* < 0.01, *** *p* < 0.001, **** *p* < 0.0001, ns = not significant. In Figure 3, *p*-value symbols directly above bars were obtained by comparing chronic or intermittent hypoxia to normoxia. The *p*-value symbols indicated above horizontal lines indicate the value obtained when comparing chronic hypoxia to intermittent hypoxia. In Figure 4, horizontal lines with P-value symbols indicate the two bars used for comparison (scrambled siRNA vs *HIF1A* siRNA). In Figure 5, the P-value symbol, ns, indicates no significance when comparing values for intermittent hypoxia and the averaged 3% hypoxia. The horizontal bar indicates the p-value symbol for comparing chronic hypoxia and averaged 3% hypoxia. In Figure 6, horizontal lines with *p*-value symbols indicate the two bars used for comparison (normox vs intermit, normox vs chronic, and chronic vs intermit).

## Abbreviations

ADM	Adrenomedullin
ANGPTL4	Angiopoietin-like 4
BHLHE40	Class E basic helix-loop-helix protein 40, also known as Dec1
BNIP3	BCL2/adenovirus E1B 19 kDa protein-interacting protein 3
CA9	Carbonic anhydrase IX
CXCL8	Interleukin 8
DDIT4	DNA-damage-inducible transcript 4
EGLN1	Prolyl hydroxylase 2 (PHD2)
EGLN3	Prolyl hydroxylase 3 (PHD3)
HIF-1	Hypoxia inducible factor-1
HK2	Hexokinase 2
LDHA	Lactate dehydrogenase A
PGK1	Phosphoglycerate kinase 1
PLOD2	Procollagen-Lysine,2-Oxoglutarate 5-Dioxygenase 2
P4HA1	Prolyl 4-Hydroxylase Subunit Alpha 1
P4HA2	Prolyl 4-Hydroxylase Subunit Alpha 2
SLC2A1	Glucose transporter 1
